# Short-Term Effect of a Health Promotion Intervention Based on the Electronic 12-Hour Dietary Recall (e-12HR) Smartphone App on Adherence to the Mediterranean Diet Among Spanish Primary Care Professionals: Randomized Controlled Clinical Trial

**DOI:** 10.2196/49302

**Published:** 2024-01-08

**Authors:** Luis María Béjar, Pedro Mesa-Rodríguez, María Dolores García-Perea

**Affiliations:** 1 Department of Preventive Medicine and Public Health Institute of Anatomy School of Medicine, University of Seville Seville Spain; 2 Camas Health Center Seville Spain; 3 Virgen Macarena University Hospital Seville Spain

**Keywords:** primary care professionals, Mediterranean diet, smartphone applications, smartphone apps, health promotion, Mediterranean diet adherence, food group

## Abstract

**Background:**

The World Health Organization has called for addressing the growing burden of noncommunicable diseases (NCDs) by promoting healthy lifestyles among the population. Regarding patient health, primary care professionals (PCPs) are the first line of care who can positively influence patients’ behavior and lifestyle habits. However, a significant percentage of PCPs do not lead a healthy lifestyle. Therefore, addressing their health behaviors may be the key to substantially increasing health promotion advice in general practice. The Mediterranean diet has been extensively studied, and there is strong evidence of it being a dietary pattern for the prevention of NCDs, in addition to its significant environmental, sociocultural, and local economics benefits.

**Objective:**

This study focused only on the dietary aspect of the PCPs’ lifestyle. The primary objective was to evaluate the effect of using the Electronic 12-Hour Dietary Recall (e-12HR) smartphone app to improve diet, specifically to promote adherence to the Mediterranean diet (AMD), among PCPs. The secondary objectives were to establish the usability of the e-12HR app and to determine AMD among PCPs.

**Methods:**

An individual-level randomized, controlled, and single-blind clinical trial was conducted with 2 parallel groups: a control group (CG), using the nonfeedback version of the e-12HR app, and an intervention group (IG), using the feedback version of the e-12HR app. The level of human involvement was fully automated through the use of the app. There was a 28-day follow-up period. Participants were PCPs (medicine or nursing) recruited offline at one of the selected primary care centers (Andalusia, Spain, Southern Europe), of both sexes, over 18 years old, possessing a smartphone, and having smartphone literacy.

**Results:**

The study response rate was 73% (71 of 97 PCPs), with 27 (38%) women and 44 (62%) men: 40 (56%) PCPs in the CG and 31 (44%) in the IG. At baseline, AMD was medium (mean Mediterranean Diet Serving Score [MDSS] index 9.45, range 0-24), with 47 (66%) PCPs with a medium/high MDSS index. There were significant statistical improvements (CG vs IG, in favor of the IG) at week 4 (no significant statistical differences at baseline): +25.6% for the MDSS index (*P*=.002) and +213.1% for the percentage with a medium/high MDSS index (*P*=.001). In relation to specific food groups, there were significant statistical improvements for fruits (+33.8%, *P*=.02), vegetables (+352%, *P*=.001), nuts (+184%, *P*=.02), and legumes (+75.1%, *P*=.03). The responses to the usability rating questionnaire were satisfactory.

**Conclusions:**

The results support recommending the use of the e-12HR app as a tool to contribute to improving diet and preventing NCDs among PCPs, while positively influencing patient dietary behavior and preventing diet-related NCDs among patients.

**Trial Registration:**

ClinicalTrials.gov NCT05532137; https://clinicaltrials.gov/study/NCT05532137

## Introduction

Major chronic noncommunicable diseases (NCDs; eg, cardiovascular diseases, cancer, chronic respiratory diseases, and diabetes) are responsible for 74% of deaths worldwide, making them the leading cause of preventable mortality [1]; they also reduce patients’ well-being and activity, which contributes to a poor quality of life, disability, and reduced productivity [1]. Notably, these NCDs share common risk factors, such as an unhealthy diet, smoking, harmful alcohol consumption, and physical inactivity [1], all of which are behavioral or lifestyle-related factors that are potentially modifiable [2]. For this reason, the World Health Organization has called for addressing the growing burden of NCDs by promoting healthy lifestyles [1,3].

Health care workers are a subgroup of the population where lifestyle promotion is essential for 3 main reasons. The first reason is their own health: although health care professionals do their best to provide exceptional patient care, they often fail to prioritize their own self-care [4], resulting in unhealthy behaviors associated with a high risk of NCDs. Studies of health care workers’ lifestyles in hospitals, for example, showed high rates of overweight/obesity [5-7], low fruit and vegetable consumption [6,7], low physical activity [5-8], tobacco [5,7] and alcohol consumption [6,7], and high levels of stress and insufficient rest [4-6]. In addition, almost half had more than 2 of these risk factors [5,6].

The second reason is the success of health care organizations: health care workers with better and healthier habits have been shown to have higher personal and job satisfaction and fewer sickness-related absences from work [9]. The third reason is patient health: several studies have described that health care professionals who practice healthy behaviors offer more advice on healthy habits to patients who come to their practice, that they have more assertive attitudes when counselling patients, and they provide more comprehensive and aggressive counselling, which can positively influence patients’ health [10-14].

Regarding patient health, primary care is the cornerstone for preventing NCDs through health education and plays an essential role in the success of therapeutic medicine [11]. Primary care professionals (PCPs) are the first line of care who can positively influence patient behavior and lifestyle habits [15], and indeed, patients perceive PCPs (doctors and nurses) as the most trusted source of health information and advice on healthy lifestyles [13]. However, a significant percentage of PCPs, as with hospital health care professionals, do not lead a healthy lifestyle: there is a high prevalence of NCD risk factors, such as smoking [10,16], alcohol abuse [10,16], overweight/obesity [17], physical inactivity [5,10,17], inadequate fruit and vegetable intake [10,11,17], added salt intake [17], and high consumption of ultraprocessed foods [18], sugars, and fats [10]. Therefore, addressing PCPs’ health behaviors may be the key to substantially increasing health promotion advice in general practice [12].

As the evidence shows, PCPs are exposed to several risk factors of NCDs; this study focused only on the dietary aspect of the PCPs’ lifestyle. As a starting point, the research team posed the following question: How can the dietary habits of PCPs be improved?

The research team has previously evaluated the effectiveness of a smartphone app called the Electronic 12-Hour Dietary Recall (e-12HR) in improving diet in Spanish university students (health science [19,20] and non–health science [20]). The main hypothesis of this study was that the use of this app among PCPs can have an influence on improving their diet, as has already been evidenced in previous research among university students [19,20], the null hypothesis being that the use of the app has no influence on improving the diet of PCPs.

To the best of our knowledge, this study is the first to evaluate the use of a smartphone app to improve diet among PCPs. The primary study objective was to evaluate the effect of using the e-12HR app on improving diet among PCPs, specifically to promote adherence to the Mediterranean diet (AMD). The Mediterranean diet has been extensively studied, and there is strong evidence of it being a dietary pattern for the prevention of NCDs [21-25], in addition to its significant environmental, sociocultural, and local economics benefits [26-28]. In addition, secondary objectives were to establish the usability of the e-12HR app and determine AMD among PCPs.

## Methods

### Overview of the Study

The study was an individual-level randomized, controlled, and single-blind clinical trial with 2 parallel groups: a control group (CG) and an intervention group (IG). All participants used an app called the e-12HR app, with different versions for the CG and the IG. In the CG, participants used the nonfeedback version of the e-12HR app, and in the IG, participants used the feedback version of the e-12HR app (see the *Intervention* section). The level of human involvement was fully automated through the use of the app.

All research was carried out in 3 basic health zones of the Andalusian Health Service (Andalusia, Spain, South of Europe): Camas, Coria del Río, and San Juan de Aznalfarache. Several primary care centers were selected in the 3 zones: the Camas, Santiponce, Valencina de la Concepción, Castilleja de Guzmán, Coca de la Piñera, Carambolo, and Pañoleta health centers in Camas; the Coria del Río health center in Coria del Río; and the San Juan de Aznalfarache and Gelves health centers in San Juan de Aznalfarache. The study ran for 28 days, and participant recruitment took place offline from September to October 2022.

Inclusion criteria for the study were both sexes, age over 18 years, possession of a smartphone (iOS or Android operating system), smartphone literacy, and a PCP (medicine or nursing) at one of the selected primary care centers. Exclusion criteria included food intolerance, chronic disease, or pregnancy (due to the possibility of requiring specific dietary recommendations).

### Ethical Considerations

The study was conducted according to the guidelines laid down in the Declaration of Helsinki, and all procedures involving study participants were approved by the Andalusian Biomedical Research Ethics Portal (PEIBA) on March 30, 2022 (identifier: 2813-N-21). The trial was registered at ClinicalTrials (identifier: NCT05532137). Written informed consent was obtained from all participants.

### Participant Enrollment

The project was publicized in the selected primary care centers by a member of the research team, and individual talks were scheduled with interested health care professionals. In each of the individual talks, the study protocol was explained, including the objectives, risks, and benefits of the research, and an email address for the study was provided.

To participate in the study, it was necessary for interested health care professionals to send an email to the designated address, indicating their “interest in participating in the study” and the primary care center where they worked. After receiving the email, a member of the research group sent candidates a series of documents necessary to be able to participate in the study: (1) an informed consent form; (2) a form with personal information (sex, date of birth, primary care center, weight, height, and smoking status), with documents 1 and 2 to be completed, signed, and returned to the same email address; (3) instructions for downloading the e-12HR app (free to download from the App Store or the Play Store); (4) an image of the Mediterranean diet pyramid (with recommendations for consumption by food group); (5) a personal alphanumeric code; and (6) a user’s guide with detailed information for using the app. Document 6 was the only one that differed depending on whether it was intended for participants in the CG (nonfeedback e-12HR version) or the IG (feedback e-12HR version), and obviously, document 5, which contained the personal code, was unique for each participant.

Throughout the study, participants could contact the research team by email with any questions, including questions to reduce the likelihood of harm.

### Participant Allocation

In each basic health zone, the recruited participants were randomized into 1 of 2 groups (CG or IG) in a ratio of 1:1 as follows: the participant who sent the first email was assigned to the CG, the participant who sent the second email was assigned to the IG, and so on.

This study was single-blind because, due to the nature of the intervention, the PCPs could not be blinded. However, the investigator who performed the statistical analysis of the data was blinded throughout the study. In addition, each participant only had access to 1 version of the app: the personal codes of the participants assigned to the CG activated only the nonfeedback e-12HR version, while the personal codes of the participants assigned to the IG activated only the feedback e-12HR version.

### Intervention

The structure and functions of the e-12HR app (nonfeedback and feedback versions) have been described in detail by Béjar et al [19]. In this study, we used e-12HR version 3.0. The e-12HR app did not undergo changes throughout the study. In brief, the nonfeedback e-12HR version allows the user to collect food consumption data; however, this version does not provide any feedback to users to promote the Mediterranean diet (ie, this version of the app presents a single function: diet determination). The feedback e-12HR version allows for the collection of food consumption data, and as an additional automatic function, every 7 days the app issues personalized feedback on how to improve AMD (ie, this version of the app has 2 functions: determining the diet and providing feedback to improve AMD). The feedback provided came in 3 parts: (1) the AMD index score: specifically, the Mediterranean Diet Serving Score (MDSS) index [29] (range 0-24); (2) the image of a traffic light: the MDSS index score was divided into 1 of 3 levels (low: score 0-8, red light; medium: score 9-15, orange light; high: score 16-24, green light) [30]; and (3) recommendations for consumption by food group [19]. The nonfeedback e-12HR version did not provide any of the 3 parts of the feedback, as they were exclusive to the e-12HR feedback version. See Multimedia Appendix 1 for real images of the e-12HR app (nonfeedback and feedback versions).

### Follow-up and Outcome Measures

To analyze the effect of the intervention (CG nonfeedback e-12HR vs IG feedback e-12HR), 4 follow-up points were established: week 1 (baseline), week 2, week 3, and week 4. At each follow-up point, the research team manually calculated the MDSS index for each of the 2 groups from the data provided by the e-12HR app. The method for calculating the MDSS index has been described in detail elsewhere [19].

The main result variable was the change in the total MDSS index at weeks 2, 3, and 4 of monitoring, while the secondary result variables were the personal information variables, the MDSS index at week 1 (baseline), and the answers to the usability rating questionnaire for the e-12HR app (see the *Usability Rating Questionnaire for the e-12HR* section).

The MDSS index at week 1 (baseline) was used to determine AMD among the PCPs (a secondary objective of the study). To relativize the data, the MDSS index of the PCPs was compared to the MDSS index of health sciences students. For a proper comparison, the MDSS index was obtained using the same app (e-12HR) and during the same follow-up period (recruitment period: September-October 2022) for both students and health care professionals.

### Usability Rating Questionnaire for the e-12HR

After the 4-week study period, a member of the research team sent a new email to each PCP who had completed the follow-up; this new email contained a usability rating questionnaire for the e-12HR app [19] (see Multimedia Appendix 2).

### Statistical Analysis

The sample size was estimated for the main result variable. Assuming SD=2.7 points, dropout rate=20.6% (from a previous study on use of the e-12HR app among health science university students [19]), α=.05, and β=.20 (bilateral test), 82 participants (n=41, 50%, per group) were needed to detect an increase of 2 points in the MDSS index (CG versus IG). The sample size was calculated using nQuery Advisor Release 7.0 (Statsols).

Quantitative variables were expressed as means (SD), and qualitative variables were displayed as numbers (percentages). The nonparametric Kolmogorov-Smirnov test was used for the test for normality.

For unpaired samples and quantitative variables, the Student *t* test or the nonparametric Mann-Whitney *U* test was used, and the chi-square test (or Fisher exact test) was used for the comparison of proportions.

For paired samples, quantitative variables, and two groups, the Student *t* test or the nonparametric Wilcoxon test was used, penalizing *P* values with Bonferroni adjustment for multiple comparisons. For 3 or more groups, the ANOVA test or the nonparametric Friedman test was used.

*P*<.05 was considered significant, except for multiple comparisons using Bonferroni penalization: *P*<.02 (.05/3).

All statistical analyses were performed using the SPSS statistical software package version 26.0 (SPSS Inc).

## Results

### Sample and Adherence to the Study

**Figure 1:**
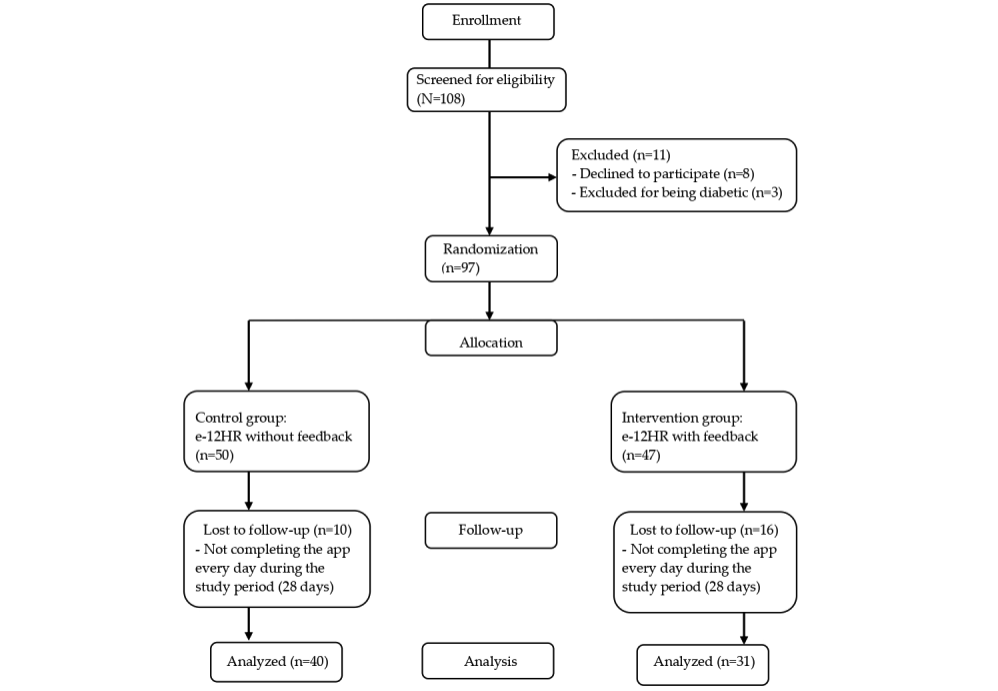
Flowchart of the study. e-12HR: Electronic 12-Hour Dietary Recall.

The sequence of allocating participants to the 2 study groups is detailed in Figure 1. In total, 97 PCPs signed the informed consent form (n=50, 52%, in the CG and n=47, 48%, in the IG). Of them, 26 (27%; n=10, 38%, in the CG and n=16, 62%, in the IG) were considered nonresponsive because they did not complete the study’s 4-week follow-up period (Figure 1). The nonresponsive individuals were not included in the later statistical analysis (ie, in this study, per protocol analysis was applied).

The study response rate was 73% (71 of 97 participants), with 40 of 50 (80%) participants in the CG and 31 of 47 (66%) participants in the IG (Figure 1). Participants did not report any harm or unintended effects throughout the study.

### Personal Information of the Participants

Table 1 shows the personal information of the PCPs who completed the study (CG and IG).

**Table 1:** Characteristics of participants who completed the study on the short-term effects of a health promotion intervention based on the e-12HR^a^ smartphone app on AMD^b^ among Spanish PCPs^c^.

Characteristics			All participants (N=71)		CG^d^ (n=40)		IG^e^ (n=31)	*P* value^f^
**Age (years)**								
	Mean (SD)	43.2 (11.6)		45.1 (11.1)		40.7 (12.1)		.07^g^
	<40, n (%)	34 (47.9)		16 (40.0)		18 (58.1)		.13^h^
	≥40, n (%)	37 (52.1)		24 (60.0)		13 (41.9)		—^i^
**Sex, n (%)**								
	Female	27 (38.0)		16 (40.0)		11 (35.5)		.70^h^
	Male	44 (62.0)		24 (60.0)		20 (64.5)		—
**BMI (kg/m^2^)**								
	Mean (SD)	25.0 (4.0)		25.3 (4.4)		24.7 (3.4)		.76^g^
	<25, n (%)	41 (57.7)		23 (57.5)		18 (58.1)		.96^h^
	≥25, n (%)	30 (42.3)		17 (42.5)		13 (41.9)		—
**Smoking status, n (%)**								
	No	64 (90.1)		37 (92.5)		27 (87.1)		.45^h^
	Yes	7 (9.9)		3 (7.5)		4 (12.9)		—
**Physical activity status (minutes/week), n (%)**								
	≥150	45 (63.4)		24 (60.0)		21 (67.7)		.50^h^
	<150	26 (36.6)		16 (40.0)		10 (32.3)		—

^a^e-12HR: Electronic 12-Hour Dietary Recall.

^b^AMD: adherence to the Mediterranean diet.

^c^PCP: primary care professional.

^d^CG: control group.

^e^IG: intervention group.

^f^*P*<.05 considered significant.

^g^Evaluated with the Mann-Whitney *U* test.

^h^Evaluated with the chi-square test.

^i^Not applicable.

No significant statistical differences were observed in the personal variables studied between the CG and the IG (Table 1). PCPs (n=47, 66%, doctors and n=24, 34%, nurses) recorded their daily consumption using the e-12HR app for a total of 1988 days (N=71 participants × 28 recording days). The app differentiated 19 food groups. Thus, a total of 37,772 daily food group consumption data points were collected during the study.

There were no significant statistical differences in the personal variables between responsive (those who completed the study) and nonresponsive (those who did not complete the study) participants.

### MDSS Index

As previously mentioned, the MDSS index was calculated manually by the research team (CG and IG, weeks 1, 2, 3, and 4) [19]. During the process, the research team corrected obvious errors made by the PCPs: for example, when the app asked for the number of standard servings of a certain food group consumed on the current day, if the participant entered a value of 150, it was considered that the data referred to milliliters or grams (instead of standard servings). In any case, only 0.2% (91 of 37,772) of the recorded data were corrected.

At week 1 of the monitoring period (baseline), that is, before the IG received the first feedback from the e-12HR app (only week 1 was considered because the feedback for IG in weeks 2, 3, and 4 could affect the alteration of the usual dietary intake), the PCPs had a mean MDSS index of 9.45 (SD 2.32), which corresponds to a medium level of adherence [30]; moreover, two-thirds of them (n=47, 66%) had a medium/high MDSS value (≥9) at baseline (week 1) [30].

#### Effect of the Intervention With the e-12HR App in Terms of Variation in the MDSS Index and Number of Participants With Medium/High (≥12) MDSS Index

Tables 2 and 3 show the MDSS index, and Table 2 also shows the number of participants with a medium/high MDSS index (≥12) in the CG and the IG throughout the 4 weeks of follow-up. We decided to use the value of 12 (instead of 9) [30] due to the high percentage of PCPs (n=47, 66%) who at baseline (week 1) already had an MDSS index≥9; therefore, using the value of 9 would have made it difficult to observe statistically significant differences between the CG and the IG.

**Table 2:** MDSS^a^ index for the CG^b^ and the IG^c^ and number of participants with a medium/high (≥12) MDSS index throughout the 4 weeks of follow-up.

Variables and week number		CG (n=40)	IG (n=31)	*P* value^d^
**MDSS index, mean (SD)**				
	Week 1	9.30 (2.40)	9.65 (2.23)	.54^e^
	Week 2	8.98 (2.84)	10.81 (2.82)	.009^e^
	Week 3	9.08 (2.45)	10.94 (3.05)	.008^e^
	Week 4	9.30 (2.59)	11.68 (3.61)	.002^f^
	*P* value^g^	.99	.01	—^h^
**Participants with a medium/high (≥12) MDSS index, n (%)**				
	Week 1	10 (25.0)	10 (32.3)	.50
	Week 2	7 (17.5)	13 (41.9)	.02
	Week 3	5 (12.5)	13 (41.9)	.005
	Week 4	7 (17.5)	17 (54.8)	.001
	*P* value	.41	.07	—

^a^MDSS: Mediterranean Diet Serving Score.

^b^CG: control group.

^c^IG: intervention group.

^d^*P* value in columns: MDSS index—intragroup differences (CG and IG) throughout the 4 weeks of follow-up in the study, evaluated with the Friedman test; number of participants with a medium/high (≥12) MDSS index—intragroup differences (CG and IG) in week 1 versus week 4, evaluated with the chi-square test. *P*<.05 was considered significant.

^e^Evaluated with the Student *t* test.

^f^Evaluated with the Mann-Whitney *U* test.

^g^*P* value in rows: MDSS index—intergroup differences (CG versus IG) in each of the 4 study weeks; number of participants with a medium/high (≥12) MDSS index—intergroup differences (CG versus IG) in each of the 4 study weeks, evaluated with the chi-square test. *P*<.05 was considered significant.

^h^Not applicable.

**Table 3:** Comparison of the MDSS^a^ index in weeks 2, 3, and 4 of follow-up with that in week 1 (baseline) for the CG^b^ and the IG^c^.

Group and week		MDSS index, mean (SD)	*P* value^d^
CG			
	Week 1	9.30 (2.40)	Reference
	Week 2	8.98 (2.84)	.34^e^
	Week 3	9.08 (2.45)	.58^e^
	Week 4	9.30 (2.59)	.99^f^
IG			
	Week 1	9.65 (2.23)	Reference
	Week 2	10.81 (2.82)	.005^f^
	Week 3	10.94 (3.05)	.004^e^
	Week 4	11.68 (3.61)	.001^f^

^a^MDSS: Mediterranean Diet Serving Score.

^b^CG: control group.

^c^IG: intervention group.

^d^Intragroup differences (CG and IG) in week 1 versus weeks 2, 3, and 4. *P*<.02 (.05/3) was considered significant (penalizing *P* values with Bonferroni adjustment for multiple comparisons).

^e^Evaluated with the Wilcoxon test.

^f^Evaluated with the Student *t* test.

Regarding intragroup modifications, there were no significant statistical differences in the MDSS index in the CG, either throughout the 4 weeks of study, ranging from mean 9.30 (SD 2.59) in weeks 1 and 4 to mean 8.98 (SD 2.84) in week 2 (Table 2) or in weeks 2, 3, and 4 when compared to week 1 (baseline; Table 3). In the IG, there were significant statistical differences in the MDSS index throughout the 4 weeks of study, ranging from mean 9.65 (2.23) in week 1 to mean 11.68 (SD 3.61) in week 4 (Table 2). Compared to week 1 (baseline), the differences were statistically significant from week 2 onward: with 1.16, 1.29, and 2.03 points of improvement at weeks 2, 3, and 4, respectively (Table 3). There were no significant statistical differences in the number of participants with a medium/high (≥12) MDSS index (week 1 vs week 4) in either the CG or the IG.

Regarding intergroup modifications, there were significant statistical differences for both the MDSS index and the number of participants with a medium/high (≥12) MDSS index in the CG versus the IG (in favor of the IG) from week 2 onward (no significant differences in week 1). For the MDSS index, we found 1.83, 1.86, and 2.38 points of improvement at weeks 2, 3, and 4, respectively; for the number of participants with a medium/high (≥12) MDSS index, we found 24.4, 29.4, and 37.3 percentage points of improvement at weeks 2, 3, and 4, respectively (Table 2).

#### Effect of the Intervention With the e-12HR App in Terms of Variation in Food Groups

Table 4 shows the number of participants who met the consumption criteria for each food group [19] in the CG and the IG throughout the 4 weeks of follow-up.

**Table 4:** Number of participants who met the consumption criteria of the MDSS^a^ index for each food group throughout the 4 weeks of follow-up (CG^b^ n=40, IG^c^ n=31).

Food group MDSS index consumption criteria and study group		Week					*P* value^d^
		Week 1	Week 2	Week 3	Week 4		
**Fruits (1-6 servings/day)**							
	CG, n (%)	26 (65.0)	27 (67.5)	27 (67.5)	27 (67.5)	.81^e^	
	IG, n (%)	24 (77.4)	27 (87.1)	28 (90.3)	28 (90.3)	.17^e^	
	*P* value^f^	.26^g^	.06^g^	.02^g^	.02^g^	—^h^	
**Vegetables (≥2 servings/day)**							
	CG, n (%)	1 (2.5)	3 (7.5)	3 (7.5)	4 (10.0)	.36^i^	
	IG, n (%)	3 (9.7)	8 (25.8)	10 (32.3)	14 (45.2)	.002^e^	
	*P* value	.31^j^	.05^j^	.01^g^	.001^g^	—	
**Cereals (1-6 servings/day of breakfast cereals, pasta, rice, and bread)**							
	CG, n (%)	40 (100.0)	37 (92.5)	39 (97.5)	37 (92.5)	.241^i^	
	IG, n (%)	31 (100.0)	30 (96.8)	30 (96.8)	30 (96.8)	.99^i^	
	*P* value	—	.63^j^	.99^j^	.63^j^	—	
**Olive oil (1-4 servings/day)**							
	CG, n (%)	31 (77.5)	33 (82.5)	33 (82.5)	34 (85.0)	.390^e^	
	IG, n (%)	26 (83.9)	26 (83.9)	28 (90.3)	27 (87.1)	.99^h^	
	*P* value	.50^f^	.88^f^	.50^i^	.99^i^	—	
**Milk and dairy products (1-3 servings/day)**							
	CG, n (%)	33 (82.5)	32 (80.0)	32 (80.0)	33 (82.5)	.99^e^	
	IG, n (%)	26 (83.9)	27 (87.1)	27 (87.1)	28 (90.3)	.71^i^	
	*P* value	.88^g^	.43^g^	.43^g^	.50^j^	—	
**Nuts (1-2 servings/day)**							
	CG, n (%)	3 (7.5)	2 (5.0)	2 (5.0)	5 (12.5)	.71^i^	
	IG, n (%)	3 (9.7)	5 (16.1)	3 (9.7)	11 (35.5)	.02^e^	
	*P* value	.99^j^	.23^j^	.65^j^	.02^g^	—	
**Fermented beverages (0-2 serving/day of wine and beer)**							
	CG, n (%)	36 (90.0)	36 (90.0)	37 (92.5)	37 (92.5)	.99^i^	
	IG, n (%)	27 (87.1)	27 (87.1)	29 (93.5)	29 (93.5)	.67^i^	
	*P* value	.72^j^	.72^j^	.99^j^	.99^j^	—	
**Potatoes (≤3 servings/week)**							
	CG, n (%)	32 (80.0)	27 (67.5)	26 (65.0)	25 (62.5)	.08^e^	
	IG, n (%)	23 (74.2)	19 (61.3)	17 (54.8)	19 (61.3)	.28^e^	
	*P* value	.56^g^	.59^g^	.39^g^	.92^g^	—	
**Legumes (≥2 servings/week)**							
	CG, n (%)	15 (37.5)	13 (32.5)	17 (42.5)	14 (35.0)	.82^e^	
	IG, n (%)	13 (41.9)	18 (58.1)	22 (71.0)	19 (61.3)	.13^e^	
	*P* value	.70^g^	.03^g^	.02^g^	.02^g^	—	
**Eggs (2-4 servings/week)**							
	CG, n (%)	21 (52.5)	24 (60.0)	22 (55.0)	26 (65.0)	.26^e^	
	IG, n (%)	19 (61.3)	20 (64.5)	17 (54.8)	17 (54.8)	.61^e^	
	*P* value	.46^g^	.70^g^	.99^g^	.39^g^	—	
**Fish (≥2 servings/week)**							
	CG, n (%)	31 (77.5)	28 (70.0)	32 (80.0)	35 (87.5)	.24^e^	
	IG, n (%)	28 (90.3)	27 (87.1)	26 (83.9)	27 (87.1)	.99^h^	
	*P* value	.15^g^	.09^g^	.68^g^	.99^j^	—	
**White meat (2-3 servings/week)**							
	CG, n (%)	20 (50.0)	11 (27.5)	10 (25.0)	11 (27.5)	.04^e^	
	IG, n (%)	10 (32.3)	9 (29.0)	12 (38.7)	5 (16.1)	.14^e^	
	*P* value	.13^g^	.89^g^	.22^g^	.26^g^	—	
**Red meat (<2 servings/week of pork, beef, lamb, and processed meat)**							
	CG, n (%)	9 (22.5)	9 (22.5)	7 (17.5)	6 (15.0)	.39^e^	
	IG, n (%)	6 (19.4)	10 (32.3)	7 (22.6)	8 (25.8)	.54^e^	
	*P* value	.75^g^	.36^g^	.59^g^	.26^g^	—	
**Sweets (≤2 servings/week)**							
	CG, n (%)	19 (47.5)	15 (37.5)	20 (50.0)	20 (50.0)	.82^e^	
	IG, n (%)	15 (48.4)	15 (48.4)	14 (45.2)	17 (54.8)	.61^e^	
	*P* value	.94^g^	.36^g^	.69^g^	.69^g^	—	

^a^MDSS: Mediterranean Diet Serving Score.

^b^CG: control group.

^c^IG: intervention group.

^d^*P* value in columns: intergroup differences (CG vs IG) in each of the 4 study weeks. *P*<.05 was considered significant.

^e^Evaluated with the chi-square test.

^f^*P* value in rows: intragroup differences (CG versus IG) in week 1 versus week 4. *P*<.05 was considered significant.

^g^Evaluated with the chi-square test.

^h^Not applicable.

^i,j^Evaluated with the Fisher exact test.

Regarding intergroup modifications, statistically significant differences (CG vs GI) were observed throughout the study period in 4 food groups: fruits (weeks 3 and 4), vegetables (weeks 2, 3, and 4), nuts (week 4), and legumes (weeks 2, 3, and 4). In these 4 food groups, at week 4, the number of participants meeting the recommendations was higher in the IG compared to the CG, with 22.8% for fruits, 35.2% for vegetables, 23.0% for nuts, and, finally, 26.3% for legumes (Table 4).

Regarding intragroup modifications, statistically significant differences (week 1 vs week 4) were observed in the CG for white meat and in the IG for vegetables and nuts.

### Usability Rating Questionnaire for the e-12HR App

Of 71 participants, 45 (63%) returned the completed questionnaire: 25 (63%) of 40 from the CG and 20 (65%) of 31 from the IG. The responses of the 45 PCPs are shown in Table 5.

**Table 5:** Responses to the usability rating questionnaire for the e-12HR^a^ app (CG^b^ n=25, IG^c^ n=20).

Questions and groups		Answers
**1. Easy to complete (strongly agree + agree)**		
	CG, n (%)	25 (100)
	IG, n (%)	20 (100)
	*P* value^d^	—^e^
**2. Understandable questions (strongly agree + agree)**		
	CG, n (%)	24 (96)
	IG, n (%)	20 (100)
	*P* value	.99^f^
**3. Understandable feedback only for the IG (strongly agree + agree)**		
	CG, n (%)	—
	IG, n (%)	18 (90)
	*P* value	—
**4. I would be willing to complete again (strongly agree + agree)**		
	CG, n (%)	20 (80)
	IG, n (%)	12 (60)
	*P* value	0.141^g^
**5. Time to complete (≤3 minutes/day)**		
	CG, n (%)	19 (76)
	IG, n (%)	15 (75)
	*P* value	.99^f^

^a^e-12HR: Electronic 12-Hour Dietary Recall.

^b^CG: control group.

^c^IG: intervention group.

^d^Differences between subgroups. *P*<.05 was considered significant.

^e^Not applicable.

^f^Evaluated with the Fisher exact test.

^g^Evaluated with the chi-square test.

No statistically significant differences were observed for any of the questions on the questionnaire (CG vs IG). All PCPs indicated that the e-12HR app was easy to complete, and most of them responded that the app contained questions that were understandable (CG n=24, 96%; IG n=20, 100%) and that feedback was understandable (only for the IG, n=18, 90%). Furthermore, a large percentage were willing to use the app again (CG n=20, 80; IG n=12, 60%). Completion of the app could be considered to have taken 3 minutes or less (CG n=19, 76%; IG n=15, 75%).

## Discussion

### Principal Findings

In relation to the main objective, there were significant statistical differences between the 2 groups in this study. At week 4 (no significant differences in week 1, baseline), the values were higher in the IG compared to the CG by 25.6% for the MDSS index (Table 2); by 213.1 for the number of participants with a medium/high (≥12) MDSS index (Table 2); and by 33.8% for fruits, 352.0% for vegetables, 184.0% for nuts, and 75.1% for legumes for the number of participants meeting the recommendations for specific food groups (Table 4).

Regarding the secondary objectives, first, the answers to the questions of the usability rating questionnaire for the e-12HR app were satisfactory. According to the questionnaire, the daily use time of the app was about 3 minutes or less per day for most respondents (Table 5). When working with smartphone apps, usability is an important aspect to consider. According to health care professionals, there are 3 principal criteria for selecting a “nutrition and diet” app for clients/patients, which are [31] ease of use (satisfactory data were obtained in this study), free of charge, and validation (the e-12HR app is free to download and has been previously validated [32-36]). Second, at baseline (week 1), AMD for PCPs was medium (mean MDSS index 9.45, SD 2.32) and 66% of participants had a medium/high MDSS index (≥9).

### Overview

To begin with, workplace interventions are an excellent strategy to promote a healthy diet, considering that health care professionals spend long hours in their professional activity and often have 1 or more meals during their working day. At the hospital level, interventions have been implemented to facilitate access to and choice of healthy foods during the working day, such as modifying the availability of foods served in the canteen, subsidizing the cost of fresh fruits and vegetables (which are often more expensive than less healthy alternatives) [37], or implementing traffic light labeling (green: healthy; yellow: less healthy; red: unhealthy) [37,38]. In a study by Thorndike et al. [38] (in a hospital in Boston, Massachusetts, USA), their intervention also included personalized automated messages using a platform that automatically generated 2 weekly emails with feedback on previous purchases in the hospital cafeteria and lifestyle advice. Significant statistical increases were observed in green-labeled food purchases and decreases in red-labeled food purchases among the IG compared to the CG in the hospital cafeteria throughout the study period.

Thorndike et al’s [38] intervention was based on information about food eaten only in the hospital cafeteria (without considering other food consumed outside the hospital), so its scope was limited. However, to date, no interventions to promote a healthy diet among PCPs have been implemented; for example, the workplace intervention strategies discussed before would be difficult to implement in Spain because health centers are widely distributed throughout the territory and do not usually have a cafeteria or restaurant. Considering these difficulties as a possible alternative strategy, this study was the first to assess the ability of a smartphone app to improve the dietary habits among health care professionals (specifically, PCPs). Several randomized controlled clinical trials have used an app to improve AMD in Spanish adults, such as patients of health care centers (the EVIDENT II app [39,40] and the SalBi Educa Nutrition app [41]) and patients with type 2 diabetes mellitus (the EVIDENT II app [42]), but not in health care professionals.

### Comparison With Prior Work

As previously mentioned, the e-12HR app has also been evaluated among Spanish university students (health sciences and non–health sciences) [19,20]. In relation to the main objective, the results obtained by the e-12HR app among PCPs compared to university students were (1) similar for the MDSS index (the increase among PCPs was 25.6% for the MDSS index, as shown in Table 2, and among university students was 17.4% [19] or 25.7% [20]), (2) more positive for the number of participants with a medium/high MDSS index (the increase among PCPs was 213.1%, as shown in Table 2, and among university student was 61.9% [19] or 74.5% [20]), and (3) less positive regarding the number of participants meeting the recommendations for specific food groups (improvements in 4 food group among PCPs, as shown in Table 4, and in 7 food groups among Spanish university students).

Regarding the secondary objectives, first, similar results were obtained among Spanish university students for the answers to the questions of the usability rating questionnaire for the e-12HR app. Second, at baseline (week 1), the mean MDSS index of 9.45 (SD 2.32) and the number of participants with a medium/high MDSS index (66%) among PCPs (Table 1) were higher compared with the data from health sciences students [20] during the same follow-up period (mean MDSS index 7.59, SD 2.72; percentage of participants with a medium/high MDSS index=33.4%). Significant statistical differences were found (*P*<.05) for both the MDSS index, which was evaluated with the Mann-Whitney *U* test, and the number of participants with a medium/high MDSS index, which was evaluated with the chi-square test: PCPs showed an improvement of 24.5% for the MDSS index and 98.2% for the number of participants with a moderate/high MDSS index (PCPs vs health science university students). This comparison must be made with caution, since the sample of PCPs was made up of doctors and nurses and the sample of health sciences university students was made up of students from the faculties of medicine and pharmacy. In addition, in a previous study by Sentenach-Carbo et al [43] among Spanish PCPs, the number of participants with medium/high AMD was lower: 55% versus 66.2%. It should, however, be considered that the adherence index used by both studies was different: the MDSS index was used in this study, and the validated 14-point Mediterranean diet adhesion screener was used in the Prevention with Mediterranean Diet (PREDIMED) study.

### Limitations

This study presents several limitations, and the first is internal validation. This included the fact that the e-12HR app is a self-reporting method and presents the limitations inherent in this type of tool, which have been amply described [44-50]. Due to the nature of the intervention, on the one hand, only the investigator who performed the statistical analysis of the data was blinded (but not the PCPs) and, on the other hand, it was not possible to guarantee that the participants were not using another nutrition app during the study period.

Regarding external validation, the dietary program was short (4 weeks), and the long-term evolution of the study variables is unknown. In addition, the evaluation of the usability of the app was based on the responses of those participants who completed the study; however, there could be differences in the perception of usability between responsive (those who completed the study) and nonresponsive (those who did not) participants.

### Future Research

According to Recio-Rodríguez et al [40], future research related to the effectiveness of apps to improve diet should clarify the possible effects of certain factors (eg, age, gender, or educational level). Therefore, in future studies, the research team intends to evaluate the effectiveness of the e-12HR app in increasing the MDSS index in different strata of PCPs—for example, examining results according to gender, age, occupational category, and the BMI as possible moderating variables and according to technological perception and technological familiarity as possible mediating variables.

### Conclusion

At baseline, Spanish PCPs presented medium AMD (measured as the MDSS index and the number of participants with a medium/high MDSS index). Throughout the study period, in the short term, the use of the e-12HR app (an easy-to-implement and low-cost intervention) showed moderate improvements in the MDSS index and remarkable improvements in the number of participants with a medium/high MDSS index; in addition, PCPs responded positively to questions about the usability of the app. These results support recommending the use of the e-12HR app as a tool to contribute to improving diet and preventing NCDs among PCPS, which, at the same time, could positively influence patient dietary behavior and prevent diet-related NCDs among the patients. From the point of view of health care organizations, the prevention of NCDs among PCPs could, in addition, lead to higher personal and job satisfaction and fewer sickness-related absences from work; for this reason, health organizations themselves should be more involved in the recommendations to use tools such as the one analyzed in this study among their own workers.

## Appendix

Multimedia Appendix 1 Real images of the e-12HR app (nonfeedback and feedback versions). e-12HR: Electronic 12-Hour Dietary Recall.

Multimedia Appendix 2 Usability rating questionnaire for the e-12HR app. e-12HR: Electronic 12-Hour Dietary Recall.

Multimedia Appendix 3 Consort checklist.

## Abbreviations

AMD: adherence to the Mediterranean diet
CG: control group
e-12HR: Electronic 12-Hour Dietary Recall
IG: intervention group
MDSS: Mediterranean Diet Serving Score
NCD: noncommunicable disease
PCP: primary care professional
