# Supplementary material for: Short-Term Effect of a Health Promotion Intervention Based on the Electronic 12-Hour Dietary Recall (e-12HR) Smartphone App on Adherence to the Mediterranean Diet Among Spanish Primary Care Professionals: Randomized Controlled Clinical Trial
Source: JMIR Mhealth Uhealth. 2024 Jan 8;12:e49302. doi: 10.2196/49302 (PMC10804253; doi:10.2196/49302)

Multimedia Appendix 1. Real images of e-12HR app (‘non-feedback’ version of e-12HR).


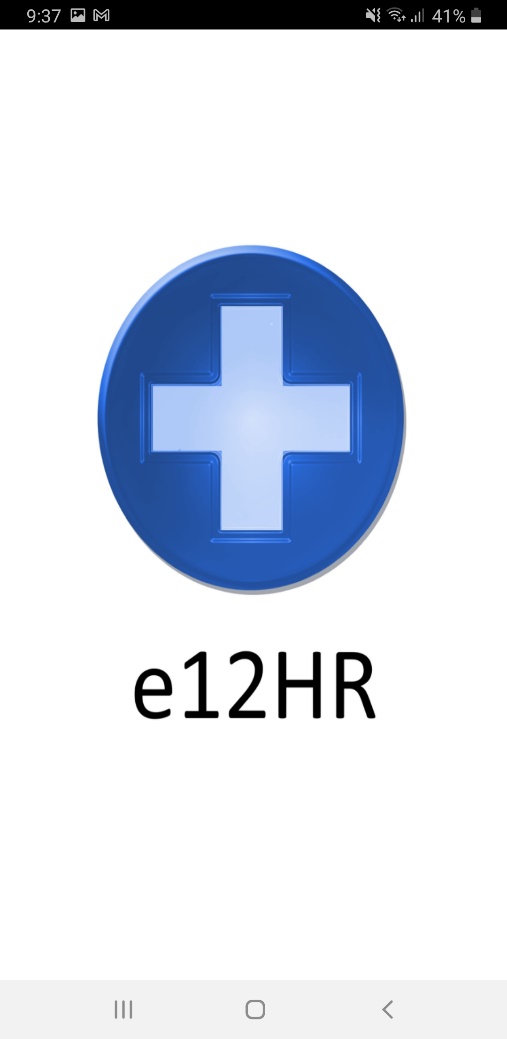

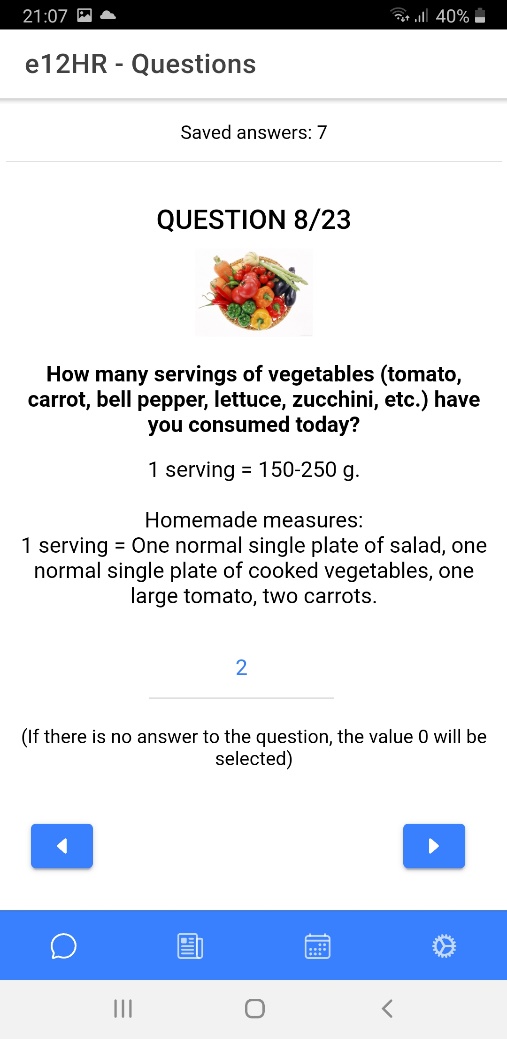

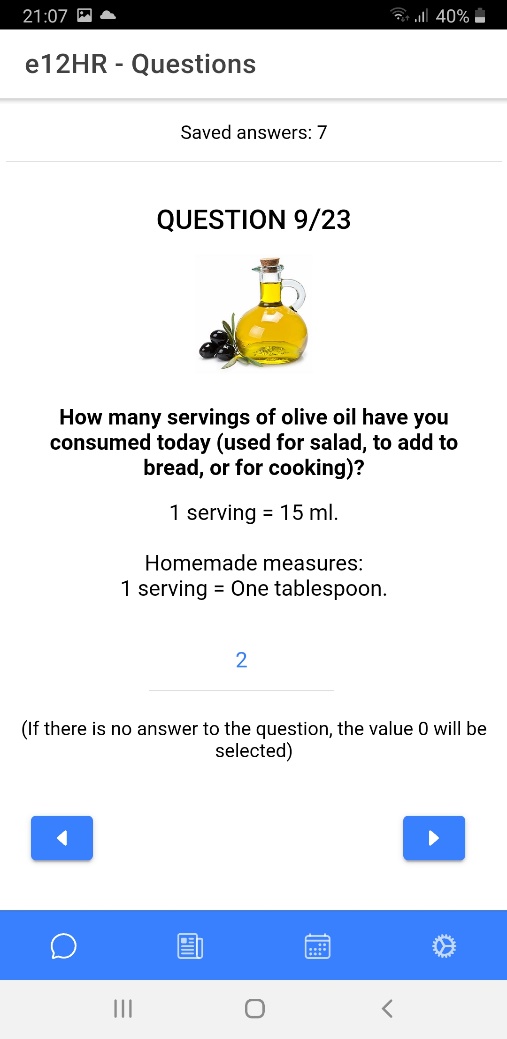


Multimedia Appendix 1. Real images of e-12HR app (‘feedback’ version of e-12HR).


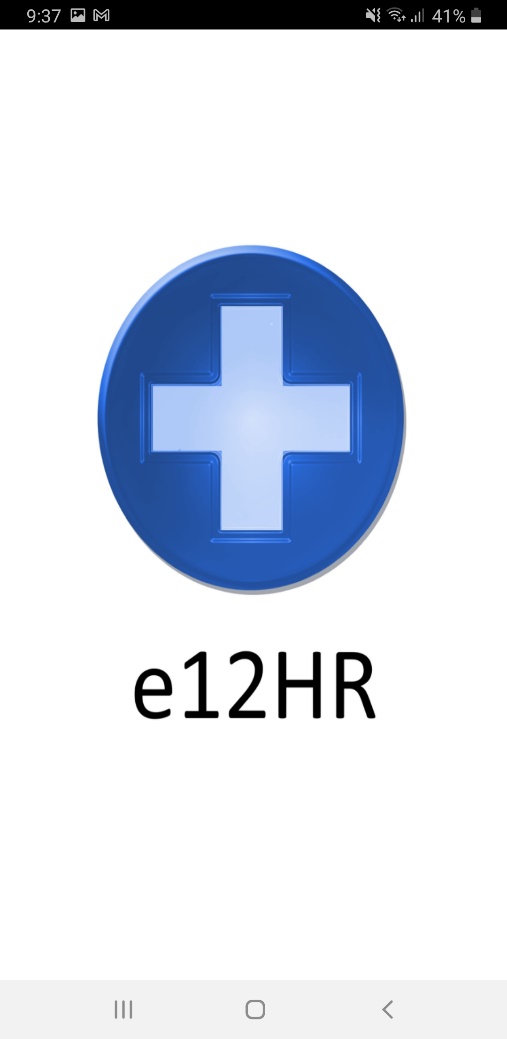

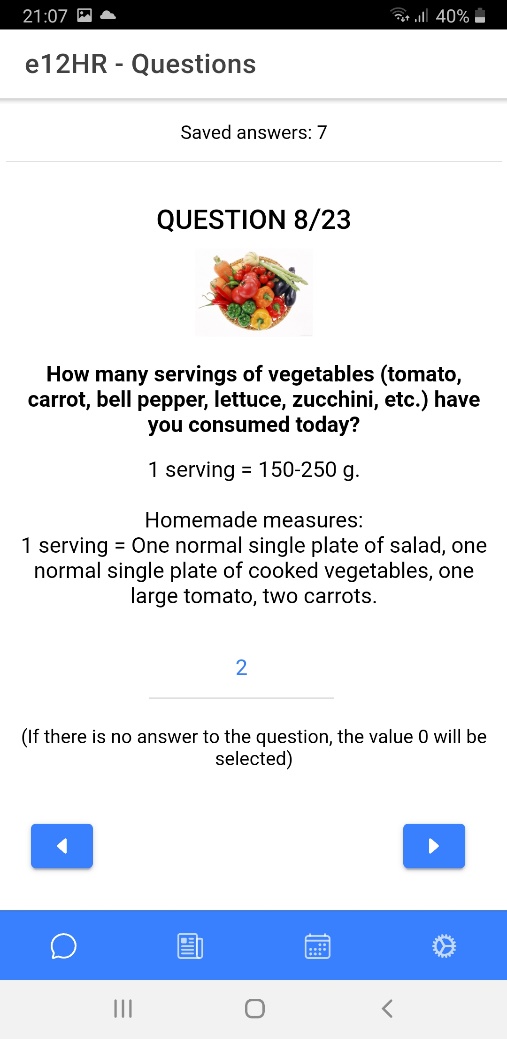

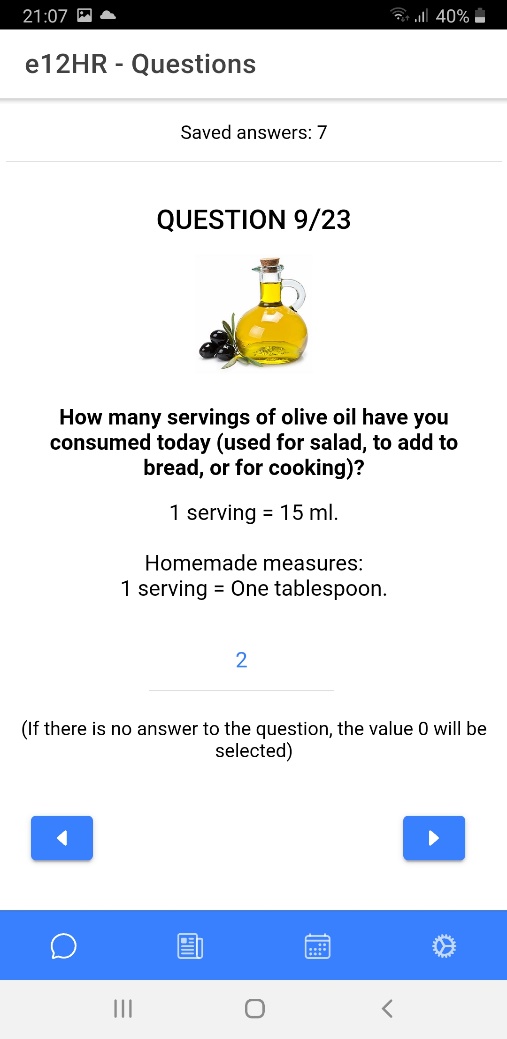


e-12HR Reports


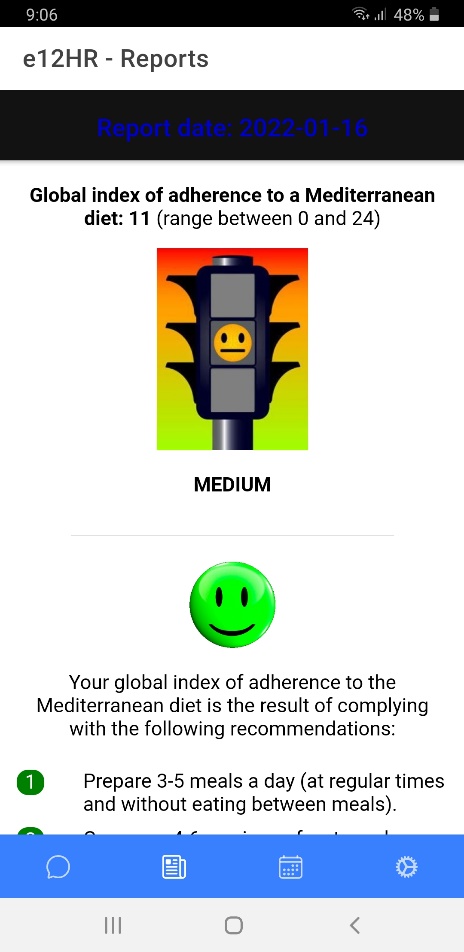

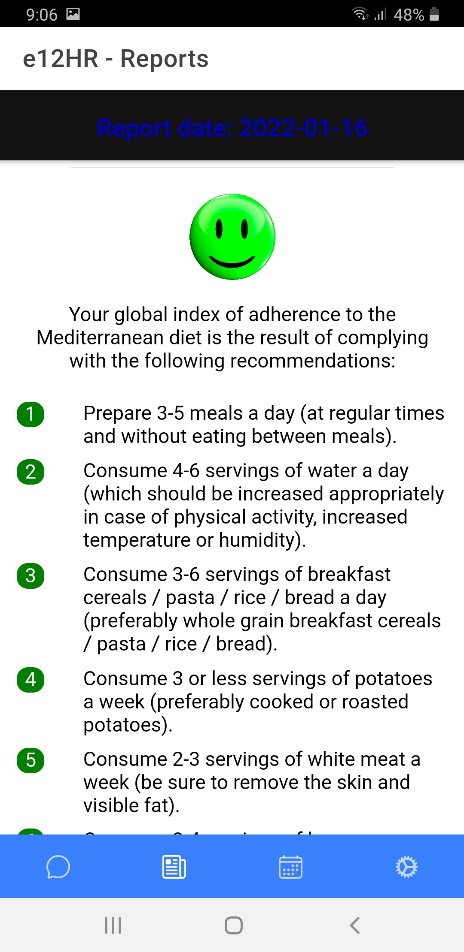

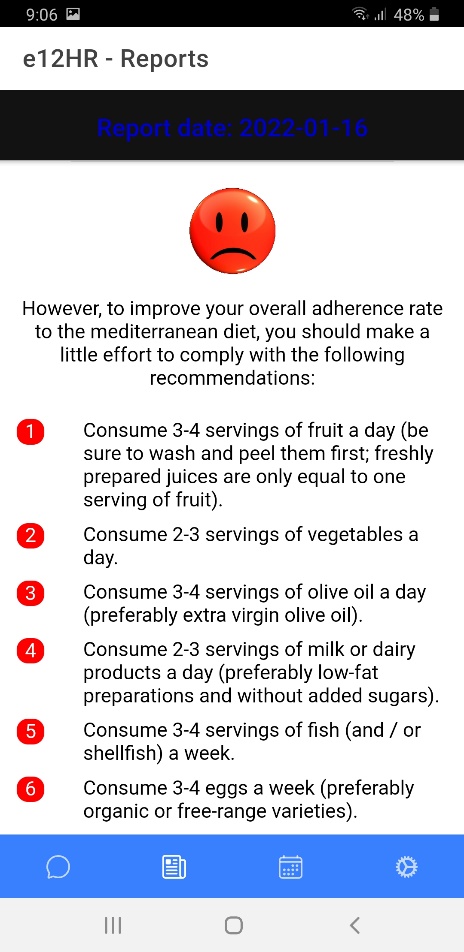

Supplement: Multimedia Appendix 1 [file mhealth_v12i1e49302_app1.docx]
