# Supplementary material for: Short-Term Effect of a Health Promotion Intervention Based on the Electronic 12-Hour Dietary Recall (e-12HR) Smartphone App on Adherence to the Mediterranean Diet Among Spanish Primary Care Professionals: Randomized Controlled Clinical Trial
Source: JMIR Mhealth Uhealth. 2024 Jan 8;12:e49302. doi: 10.2196/49302 (PMC10804253; doi:10.2196/49302)
Supplement: Multimedia Appendix 2 [file mhealth_v12i1e49302_app2.docx]

Multimedia Appendix 2. Usability rating questionnaire for e-12HR.

| **1. I found e-12HR easy to complete:** |
| --- |
| 1  2  3  4  5 |
| **2. I found the questions of e-12HR understandable:** |
| 1  2  3  4  5 |
| **3. I found the feedback from e-12HR understandable (only for intervention group):** |
| 1  2  3  4  5 |
| **4. I would be willing to complete e-12HR again:** |
| 1  2  3  4  5 |
| **5. How much time to complete the app (per day):** |
| < 1 min./day  Approx. 1 min./day  Approx. 2 min./day  Approx. 3 min./day  Approx. 4 min./day  ≥ 5 min./day |
| 1: Strongly agree.  2: Agree.  3: Neither agree nor disagree.  4: Disagree.  5: Strongly disagree. |
